# Integrating Nutrition and Stress into Life History Stages of Wild Free-Ranging Female Pronghorn

**DOI:** 10.3390/ani16142115

**Published:** 2026-07-08

**Authors:** Cole A. Bleke, Eric M. Gese, Juan J. Villalba, Shane B. Roberts, Susannah S. French

**Affiliations:** 1 Department of Wildland Resources, Utah State University, Logan, UT 84322, USA; juan.villalba@usu.edu; 2 U.S. Department of Agriculture, Wildlife Services, National Wildlife Research Center, Utah Field Station, Logan, UT 84322, USA; eric.gese@usu.edu; 3 Idaho Department of Fish and Game, Boise, ID 83712, USA; shane.roberts@idfg.idaho.gov; 4 Department of Biology and the Ecology Center, Utah State University, Logan, UT 84322, USA; susannah.french@usu.edu

**Keywords:** *Antilocapra americana*, diet quality, fecal, life history stages, nutrition, stress, pronghorn antelope

## Abstract

Studies addressing seasonal changes in diet quality and hormones are important for understanding the interactions between ecology and physiology. We collected fecal samples from free-ranging adult female pronghorn (*Antilocapra americana*) to examine the relationships between indicators of nutrition and glucocorticoid activity. This occurred during three population-level, life-history-correlated sampling periods across five subpopulations. We evaluated nutrition via fecal nitrogen, fecal 2,6-diaminopimelic acid (DAPA), and assessed glucocorticoid activity using fecal glucocorticoid metabolites (FGM). We found positive relationships between fecal nitrogen and DAPA, whereas DAPA and fecal nitrogen with FGM varied in influence and strength, depending upon life history stage. Generally, correlations between DAPA and FGM were strongest during late gestation sampling, whereas correlations between those two measures and fecal nitrogen lacked consistency across life history stages and years. We found that maternal nutrition and stress were associated with notable changes in the relative intake of dietary protein from plant functional groups across sampling periods. Pronghorn appeared to shift between diets high in nitrogen or digestible energy content, depending upon life history stage and associated metabolic demands. We feel these results will assist wildlife managers in further understanding relationships between physiological parameters and how pronghorn meet the metabolic requirements of life history stages.

## Simple Summary

Studies addressing seasonal changes in ungulate diet quality and hormones are important for understanding the connection between ecology and physiology. Life history stage likely plays a large role in nutritional needs and stress accumulation in sexually mature female pronghorn. We found that adult female pronghorn nutrition and stress vary by life history stage, associated with seasonal changes in demands and forage quality. These findings will assist wildlife managers in gaining a deeper understanding of the relationship between nutrition and stress, and of how adult female pronghorn meet these varying metabolic requirements across consecutive life history stages.

## 1. Introduction

A comprehensive understanding of population demographic metrics (e.g., abundance, survival, recruitment) is vital for managing wildlife species; however, these estimates can be expensive and difficult to obtain [1]. These methods may compromise the physiological state of the animal [2] and present potential hazards to both the animal [3] and the handler [4]. Conversely, noninvasive methods (e.g., urine and fecal collections) offer more frequent biological sampling opportunities to obtain physiological information than invasive methods (e.g., capture and handling), which often lack repetitive sampling opportunities. The use of indirect measures of diet quality (i.e., fecal indices) has become a common option for researchers, as samples are easy to collect and cause minimal disturbance to the animal [5]. Two commonly used fecal indices for assessing physiological condition are fecal nitrogen and fecal 2,6-diaminopimelic acid (DAPA [6,7]). Fecal nitrogen is positively linked to dietary nitrogen, protein content, and digestibility, and represents diet quality [5,8,9,10]. 2,6-diaminopimelic acid is a unique amino acid residue of rumen bacterial fermentation found in fecal matter, represents rumen bacterial population mass, and is a proxy for digestible energy [11]. Extensive research efforts have been directed toward understanding the nutritional ecology of ungulates to define the relationships between the environment and the use of energy and nutrients by individuals and populations, understanding spatial and temporal differences, and the influence of life history stages on diet selection, but the focus has largely been on species within the family Cervidae [12].

Nutrition refers to an individual’s or population’s biophysical status, dietary intake of foods and their components, and metabolic responses to ingested foods [13]. Monitoring the diet quality of wild ruminants is useful for management and aids in further understanding aspects of species’ ecology, in particular the dynamics of populations in relation to the quality of their food resources [12]. The quality of diets is an essential driver of individual performance, which, in turn, affects population dynamics [14]. Energy and/or protein are often the most limiting dietary factors for wild herbivores, which has led to research focused on indices designed to estimate their concentration in animal diets [9].

Acquisition of energy and nutrients is dependent on the plant growth forms available in the landscape [15]. Grasses and forbs uptake nutrients faster than shrubs in the spring, thus showing faster phenological changes [16]. Changes in diet quality associated with phenological changes could lead to nutritional stress. Stress is a cascade of neurological, hormonal, and immunological responses to changes in the environment [17]. Researchers use stress measurements as a tool to evaluate the condition of an ungulate population at specific times or locations, to monitor the relationship between animals and their environment, and to examine the influence of human disturbances on wild or domestic animal species [18]. Environmental or anthropogenic disturbances can trigger activation of the vertebrate neuroendocrine axis, often resulting in the release of stress hormones (e.g., catecholamines and glucocorticoids [GC] [19,20]). Glucocorticoid hormones are produced by the hypothalamic–pituitary–adrenal (HPA) axis and play a fundamental role in the regulation of energy homeostasis in relation to ‘predictable’ life history events such as growth, reproduction, and migration [21,22]. Glucocorticoids are also measured in stress-induced situations in which these hormones coordinate physiological and behavioral responses to unpredictable environmental changes or perceived abiotic and biotic challenges [23].

Environmental challenges (e.g., winter severity stress) that activate the HPA axis can be either acute or chronic [19], and the biological matrix (e.g., blood, saliva, excreta, integumentary structures) used to measure GCs reveals varying longitudinal measures from “free” or “bound” hormones [24]. Fecal hormones are produced via plasma GCs metabolized by the liver and excreted into the gut via bile ducts [25,26]. Fecal glucocorticoid metabolites (FGMs) reflect the free, or unbound, fraction of total glucocorticoids [27,28]. Hormone metabolites in feces (e.g., FGM) represent an integrated measure of the stress response over 12 to 24 h in ungulates [29,30], depending upon gut retention time [31].

Pronghorn antelope (*Antilocapra americana*) are a small ruminant (45 kg) native to sagebrush (*Artemisia* spp.) and grassland ecosystems in western North America [32,33]. Pronghorn have a unique distinction among mammals in that they are the only members of the family Antilocapridae and the genus *Antilocapra* [34]. The current distribution of pronghorn is but a fraction of their historic range [35], prompting research into techniques for monitoring population status. They are particularly sensitive to stress and mortality during physical capture [36], and myopathy is not an uncommon consequence of handling [37,38], making alternative methods such as noninvasive sampling appealing. The goal of our study was to investigate the relationship between nutrition and stress during peak life history stages in adult female pronghorn. While previous research has explored the relationships between fecal indicators of nutrition or stress in pronghorn [6,7,10,39,40,41], many of these studies investigated maternal nutrition or maternal stress, but not both concurrently [13]. Pronghorn diet composition was explored to investigate potential seasonal differences across plant functional groups (i.e., forb, graminoid, legume, shrub [42]) as an initial step toward integrating with nutritional indices. Thus, the objectives of our study were (1) to investigate the temporal physiological relationships between fecal nitrogen, DAPA, and FGM and (2) to assess adult female pronghorn nutritional condition and physiological stress across consecutive life history stages and among different habitats. We hypothesized that environmental and seasonal variables, including diet quality and life history stage, would collectively influence these parameters.

## 2. Materials and Methods

### 2.1. Study Area

**Figure 1:**
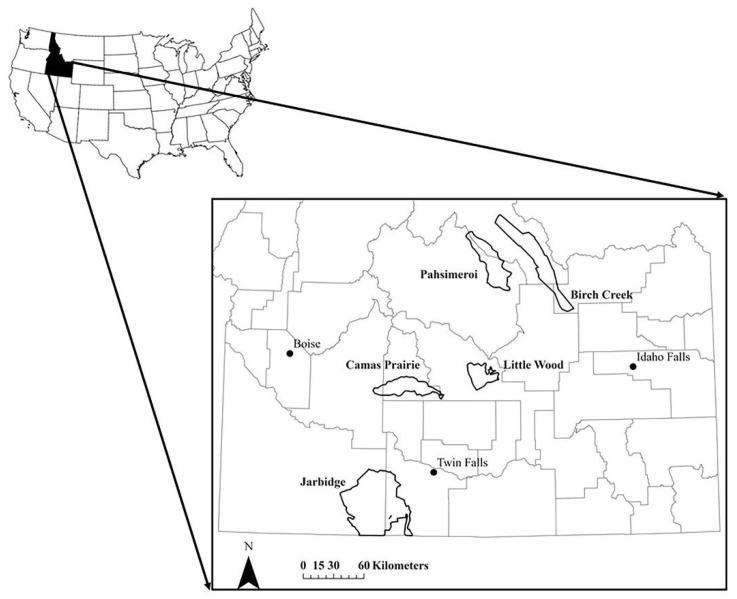
Pronghorn antelope summer distributions. Ranges of the five pronghorn antelope study subpopulations (Birch Creek, Camas Prairie, Jarbidge, Little Wood, Pahsimeroi) within the state of Idaho. Gray lines represent county boundaries.

This study involved five pronghorn subpopulations representing the majority of pronghorn habitats in southern Idaho, USA, including Jarbidge, Camas Prairie, Little Wood, Birch Creek, and Pahsimeroi [39] (Figure 1). The Jarbidge site contained a resident pronghorn subpopulation occupying desert habitat. Based on Idaho’s gap analysis land cover classification [43], basin and Wyoming big sagebrush (*Artemisia tridentata tridentata* and *A. t. wyomingensis*) were the dominant cover types. Perennial grasslands were the next dominant cover type, with the remaining landscape being a mix of low sagebrush (*Artemesia arbuscula*), antelope bitterbrush (*Purshia tridentata*), and rabbit brush (*Chrysothamnus* spp.) communities. The Camas Prairie site supported a migratory pronghorn subpopulation that largely persisted on agricultural lands during the spring and summer months. Alfalfa (*Medicago sativa*) was the dominant crop, followed by planted grassland parcels enrolled in the Conservation Reserve Program [44]. The Little Wood area also contained a migratory pronghorn subpopulation occupying native shrub-steppe rangelands with big sagebrush and irrigated agriculture throughout. The Birch Creek and Pahsimeroi study sites contained migratory subpopulations inhabiting mountain-valley habitats. The Birch Creek subpopulation occupied both the Birch Creek and Lemhi valleys, where low sagebrush was the dominant vegetation community. The mountain, basin, and Wyoming big sagebrush also accounted for a large portion of the study site, with limited agricultural lands. The Pahsimeroi subpopulation occupied both the Pahsimeroi and Little Lost River valleys, where mixed stands of mountain big sagebrush and low sagebrush dominated the landscape. Basin and Wyoming big sagebrush were the next most abundant cover types, and agricultural lands were also limited [39]. See Bleke et al. [45] for additional descriptions of habitat types.

### 2.2. Sample Collections

We randomly collected fresh fecal samples from unmarked, reproductive-aged female pronghorn (i.e., ≥2 years) in 2018 and 2019. Collections occurred during three sampling periods, selected to coincide with expected maternal life-history stage timeframes: late gestation (April to mid-May), early lactation (June), and the breeding season (September). We discuss our results at the population level and by female life history stage, because we were unable to associate pregnancy or lactation status with individual adult female pronghorn samples due to our noninvasive sampling design. We used magnifying optics (Vortex Optics, Barneveld, WI, USA) to categorize individuals by age class (i.e., fawn, yearling, adult, unknown) and sex and to monitor defecation. Females were identified by the lack of a black cheek patch. We used 2-person teams and two-way radios (Motorola Solutions, Chicago, IL, USA) to locate fresh pellet piles. If we were uncertain whether an individual female pronghorn was sexually mature (e.g., a lone individual that lacked a reference adult female for age or size comparison) or we believed an individual was a yearling based on estimated shoulder height or muzzle length, then we did not collect a sample. We collected fecal samples from spatially segregated groups of animals to obtain a representative sample of the subpopulation and avoid duplicate sampling.

### 2.3. Laboratory Methodologies

We collected a total of 1440 samples across two years, three sampling periods each year, and five subpopulations during our study. From those, we randomly selected 20 samples/subpopulation/sampling period/year (*n* = 560 samples; 2018 = 260, 2019 = 300) for analyses of fecal glucocorticoid metabolites and nutritional indices via fecal nitrogen and DAPA. The Little Wood and Camas Prairie subpopulations were not sampled during late gestation in 2018. We dried fecal samples in a drying oven (Precision Scientific, Chicago, IL, USA) at temperatures below 50 °C until all moisture evaporated. We ground dried samples with a coffee grinder (Hamilton Beach, Southern Pines, NC, USA) until the fecal material formed a consistent powder. We sent portions of the dried and ground samples to the Wildlife Habitat and Nutrition Laboratory at Washington State University (Pullman, WA, USA) to measure concentrations of DAPA and fecal nitrogen. DAPA was calculated following the methods of Davitt and Nelson [11], while fecal nitrogen percent was determined using a TruSpec CN Analyzer (LECO, St. Joseph, MI, USA). We used cortisol enzyme-linked immunosorbent assay (ELISA) kits (ADI-900-071, Enzo Life Sciences, Inc., Farmingdale, NY, USA) to measure FGM concentrations via a microplate reader (BioRad, Hercules, CA, USA). We followed established protocols that were optimized for pronghorn for fecal steroid metabolite extractions, validations, and measurements. Hormone validations were completed to determine appropriate hormone concentrations, control for assay precision, and detect any potential nonspecific binding that would bias results. We also added spikes to calculate recovery, ensuring that only the hormone of interest was measured and no binding interference occurred [45]. Additionally, we validated our preservation and homogenization methods, as described by Hadinger et al. [46]. Ideally, we would have performed an adrenocorticotropic hormone (ACTH) challenge to help confirm our FGM concentrations; however, this was not logistically possible as the study was undertaken in the field with free-ranging animals. Fecal glucocorticoid metabolite measurements were calculated in 34 assays with a mean intra-assay variation of 1.96% and an inter-assay variation of 15.42%.

### 2.4. Statistical Analysis

We used multivariate analysis of variance (MANOVA) to evaluate differences in the measured mean values of DAPA, FGM, and fecal nitrogen across subpopulations, years, and sampling periods in R version 4.1.2 [47]. We used post hoc Tukey’s honest significant difference (HSD) tests for comparisons when multivariate analyses identified statistical differences. We used Pearson’s correlation coefficients to evaluate overall and seasonal relationships among fecal nitrogen, DAPA, and FGM, with the strength of the relationship determined by a regression line and 95% confidence intervals. This calculation included samples from all five subpopulations, with overall interest at the temporal rather than the spatial scale. Samples associated with FGM concentrations were standardized by dry-weight fecal mass, and results were converted from pg/mL^−1^ to ng/g^−1^ for each sample. All mean values are reported ± standard error and used α = 0.05 for all statistical tests.

## 3. Results

### 3.1. MANOVA of Life History Stages

**Figure 2:**
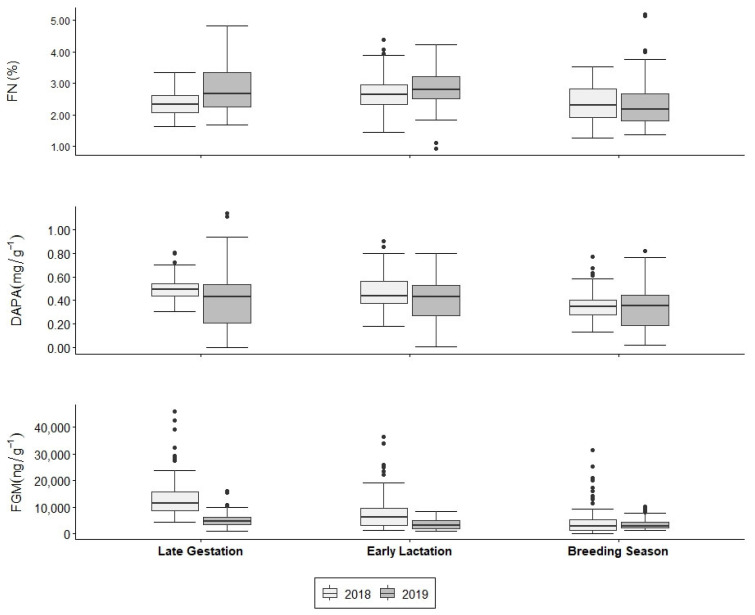
Mean values of fecal nitrogen (FN [%]), 2,6-diaminopimelic acid (DAPA [mg/g^−1^]), and fecal glucocorticoid metabolites (FGM [ng/g^−1^]) from five pronghorn subpopulations in Idaho during 2018 and 2019 sampling periods.

We found interannual differences for fecal nitrogen (F_1, 558_ = 13.15, *p* < 0.001), DAPA (F_1, 558_ = 13.92, *p* < 0.01), and FGM (F_1, 558_ = 71.36, *p* < 0.01), an intra-annual differences in fecal nitrogen (F_1, 558_ = 5.21, *p* = 0.02) and FGM (F_1, 558_ = 16.21, *p* < 0.01), as well as a subpopulation difference in DAPA (F_1, 558_ = 4.64, *p* = 0.03) and FGM (F_1, 558_ = 25.43, *p* < 0.01). We found September breeding-season fecal nitrogen to be significantly lower than June early lactation (*p*. adj. < 0.01) and April–May late gestation (*p*. adj. < 0.01). Seasonal FGM differences occurred between breeding season and early lactation (*p*. adj = 0.02), late gestation and early lactation (*p*. adj < 0.01), and late gestation and breeding season (*p*. adj < 0.01; Figure 2).

### 3.2. Correlations of Physiological Measures

**Figure 3:**
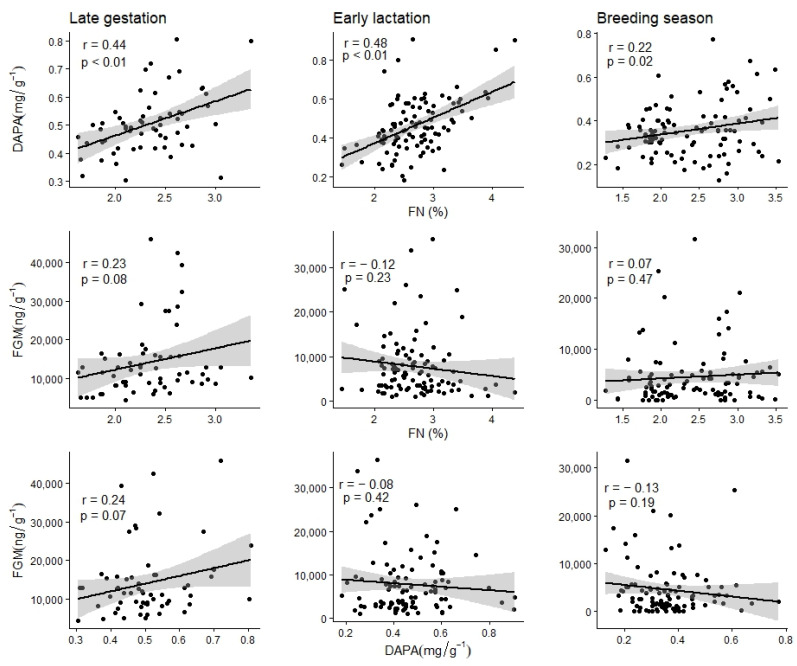
Correlations between fecal nitrogen (FN [%]), 2,6-diaminopimelic acid (DAPA [mg/g^−1^]), and fecal glucocorticoid metabolites (FGM [ng/g^−1^]) during 2018 sampling that coincided with late gestation, early lactation, and breeding season timeframes across five pronghorn subpopulations in Idaho. The black line represents the regression line, and the gray shaded area represents the 95% confidence interval.

**Figure 4:**
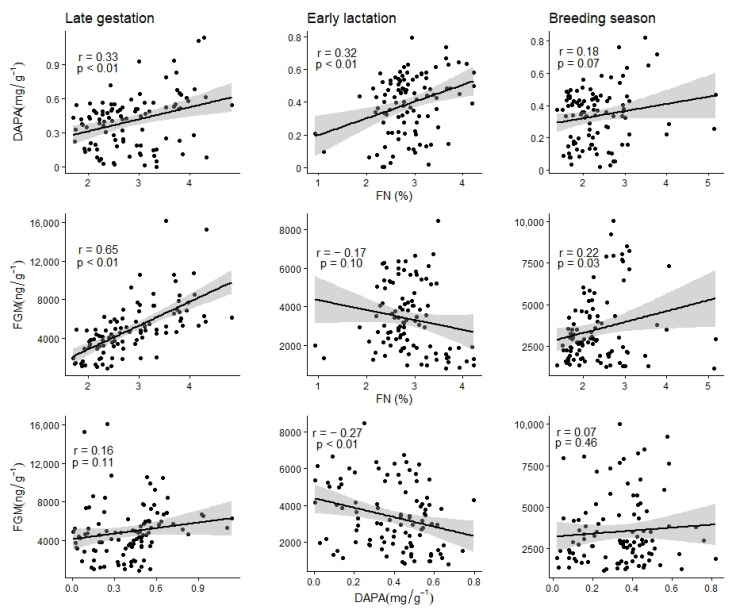
Correlations between fecal nitrogen (FN [%]), 2,6-diaminopimelic acid (DAPA [mg/g^−1^]), and fecal glucocorticoid metabolites (FGM [ng/g^−1^]) during 2019 sampling that coincided with late gestation, early lactation, and breeding season timeframes across five pronghorn subpopulations in Idaho. The black line represents the regression line, and the gray shaded area represents the 95% confidence interval.

Fecal nitrogen and DAPA correlation was highest during early lactation in 2018 (r = 0.48, df = 100, *p* < 0.01; Figure 3) and late gestation in 2019 (r = 0.33, df = 98, *p* < 0.01; Figure 4). Fecal nitrogen and FGM were positively correlated during late gestation (r = 0.65, df = 98, *p* < 0.01) and breeding season (r = 0.22, df = 98, *p* = 0.03) in 2019 (Figure 4). The strongest overall positive correlation detected occurred during late gestation in 2019 between fecal nitrogen and FGM (r = 0.65, df = 98, *p* < 0.01), while the strongest negative correlation occurred during early lactation in 2019 between DAPA and FGM (r = −0.27, df = 98, *p* < 0.01; Figure 4).

## 4. Discussion

### 4.1. Overall Life Stage Nutrition and Stress Relationships

We found annual differences in all measures across free-ranging adult female pronghorn inhabiting the shrub-steppe biome in southern Idaho. Fecal nitrogen and DAPA were positively correlated during all sampling periods (i.e., late gestation [r = 0.44], early lactation [r = 0.48], and breeding season [r = 0.22]) in 2018 as well as late gestation (r = 0.33) and early lactation (r = 0.32) sampling in 2019 (Figure 3 and Figure 4). Fecal nitrogen and FGM were positively correlated during late gestation (r = 0.65) and breeding season (r = 0.22) in 2019, while FGM and DAPA were negatively correlated during early lactation (r = −0.27) in 2019 (Figure 4). The reliability of fecal nitrogen as an index of dietary quality may become compromised by forage species with high levels of condensed tannins, without affecting DAPA levels, as this amino acid passes unabsorbed through the digestive tract with no measurable loss [11]. Thus, fecal nitrogen and DAPA together provide an accurate assessment of dietary quality even in the event of ruminants ingesting a diet high in condensed tannin content, which compromises nitrogen absorption [10]. Nevertheless, results from our concurrent plant DNA barcoding for diet analysis demonstrated that these pronghorn subpopulations consumed a large proportion of protein from low-tannin-containing forbs [42], suggesting fecal nitrogen is likely a useful measure of dietary quality [48]. The utility of fecal nitrogen and DAPA to reflect changes in nutritional plane has been demonstrated through feeding trials of captive pronghorn [7] and applied to free-ranging pronghorn to track changes in seasonal diet quality [6,39,49,50], as well as to compare diet quality between populations [50]. Food and nutrient availability are the ultimate sources of energy during the reproductive cycle of ungulates [51], but there are complex interactions between internal physiological processes and the external environment [12,52]. For instance, adult females select diets high in nutritional value during spring, summer, and autumn, in response to their highest energetic demands from late winter (late gestation) to mid-summer (lactation) in a context of high temporal and spatial variability in forage abundance and nutritional quality [12]. This study expanded the temporal window of measurements in these subpopulations relative to previous studies [39,41], allowing the assessment of when female pronghorn seek higher-quality diets. Using plant DNA barcoding results, we were able to establish the functional groups used by pronghorn for dietary protein intake [42], in contrast to these studies, which assumed selection based on the availability of forages present in the landscape, such as the presence of agricultural crops (e.g., alfalfa).

Correlations between FGM and nutritional indices varied across life-history stages each year, with the strongest positive correlations occurring during late gestation and breeding-season sampling, and the strongest negative correlation during early lactation sampling in 2019. Glucocorticoids are known to increase in the period leading up to parturition [53,54]. Early lactation sampling in 2019 showed the strongest negative correlation between nutrition and FGM; however, relationships between nutritional indices and FGM for both years were negative. This can be explained by the established relationship between FN and the normalized difference vegetation index (NDVI [48]) as well as by the likely interplay between maternal nutrition and stress [13].

### 4.2. Late Gestation

Pronghorn demonstrated a positive relationship between fecal nitrogen and DAPA during late gestation sampling in both years (Figure 3 and Figure 4). Mean fecal nitrogen values during late gestation sampling were similar to those during early lactation but greater than those during the breeding season. We found DAPA differed between years. This difference may have resulted from differences in sample size between years. Hansen et al. [50] found pronghorn DAPA to be highest in the spring. Springtime DAPA concentrations are likely linked to forb availability and the energetic demands of late gestation for protein. Pronghorn in our study appeared to consume diets higher in digestible energy than in dietary nitrogen during late gestation, sampling each year, as the relative intake of protein from graminoids was highest during this time [42]. A greater proportion of protein intake from graminoids occurred in 2018 than in 2019, potentially increasing rumen activity to extract more energy from the higher intake of dietary grasses, a functional group with a high content of lignocellulose [55]. Both plant crude protein content and digestibility peak early in the growing season, likely linked to increased protein demands in late gestation, and then decline rapidly as vegetation matures. Higher forage quality is thus associated with early phenological stages where new green leaves dominate biomass [56]. Diets that maximize energy intake during spring have also been found in red deer (*Cervus elaphus* [57]) and bison (*Bison bison* [58]).

Fecal glucocorticoid metabolite concentrations were highest during late gestation for each year, but averages differed between years (Figure 3 and Figure 4). We acknowledge that late gestation correlations may be confounded by sampling timing relative to parturition. Our late-gestation sampling occurred from April to mid-May in both years, aligning with the third trimester of gestation, the most metabolically demanding period of pregnancy in ungulates [12]. Metabolic costs during this time are 50% greater for pregnant than for nonpregnant females [51]. Pronghorn gestation ranks among the longest of North American ruminants (i.e., 250 ± 8.78 days [59,60]), and this duration is speculated to be a result of does being stressed to the limit of their capabilities to produce two large fawns [61]. Variation in gestation length is directly influenced by the nutritional condition of the gravid female [59], and twin-bearing pronghorn are believed to invest more in reproduction than all other ungulate species per reproductive event [62,63], expending considerable energetic reserves during prenatal and postnatal phases of offspring development [60,61,62]. Correlations between maternal nutrition and maternal stress were strongest during late gestation, but this is likely an artifact of increased nutritional demands during this time, as well as the seasonal influence of GCs. Glucocorticoids play an important role throughout gestation and the period leading up to parturition, acting as mediators that direct nutrients and energy to the highly specialized maternal reproductive tissues and the developing fetus [64]. Pregnancy dramatically affects the maternal HPA as circulating glucocorticoids increase from middle to late gestation [48,55]. Elevated glucocorticoids are usually interpreted as indicative of an energetically demanding event for an animal [65], but this increase is believed to also assist in multiple aspects of female survival and natal development, such as feed intake stimulation [66], fetal organ development [55], decreased cortisol sensitivity to protect the developing fetus [67], increased fat stores to support lactation, uterine contraction, expulsion of the fetus, and rejection of the placenta [68,69]. This may likely explain why we found FGM concentrations to be highest during late gestation, as well as the influence of sample collection in relation to parturition.

### 4.3. Early Lactation

Relationships between fecal nitrogen and DAPA were positively correlated during the early lactation sampling period in both years (Figure 3 and Figure 4). In addition, diet quality (i.e., fecal nitrogen) increased while digestible energy decreased from late gestation to early lactation in both years (Figure 2). Sampling occurred throughout the month of June to coincide with the early lactation period in pronghorn. The transition from pregnancy to lactation is among the most important determinants of maternal-offspring health outcomes [70,71,72]. Lactation is the biological period when daily energetic requirements are highest for female ungulate species [12], where requirements increase by 65–215% during the first month postpartum [73,74]. Substantial physiological changes occur during this transition, including changes in hormones, immune system, and metabolism, to shift resource allocation from energy storage to milk synthesis [75,76], enabling pronghorn fawns to attain adult proportions rapidly [77]. These measurements of fecal nitrogen and DAPA may also have come from females that lost offspring, did not reproduce, or were not actively lactating.

Dietary quantity and quality are highly variable, particularly in environments with prominent seasonal changes [12]. The increase in dietary nitrogen was reflected by an increase in dietary breadth and intake of protein from forbs compared to late gestation [45], aligning with the thought that the highest intakes of digestible nutrients by herbivores occur during early summer as nutrient availability in summer drives replenishment of body reserves and subsequent reproductive success [12]. Bleke et al. [48] found NDVI estimation during early lactation to be strongly correlated with fawn summer survival.

Relationships between maternal nutrition and FGM concentrations were negative during the early lactation sampling period for both years (Figure 3 and Figure 4). Our findings suggest that an improvement in female nutritional condition, as well as a successful life-history stage, results in a decline of FGM concentrations. Following parturition, maternal glucocorticoids sharply decrease in concentration yet continue to play a role in lactogenesis and milk secretion [55]. Peak milk volume and milk energy output per unit of maternal weight are greater in pronghorn than in any other studied species of North American ungulates [78]. Growth rates of neonatal pronghorn have been directly related to milk energy intake, which is highest during early lactation, and directly related to doe body condition. It is advantageous for pronghorn fawns to attain adult proportions rapidly, as the efficiency of locomotion increases with body weight and leg length. This is significant because the sooner neonates can match adult running speeds and stay with a group, the better their chance of survival and predator avoidance [32].

### 4.4. Breeding Season

Relationships between fecal nitrogen and DAPA were positive during breeding season sampling for both years (Figure 3 and Figure 4). Mean fecal nitrogen and DAPA values decreased from early lactation to breeding season as female demands for nitrogen during this time tend to be lower than during gestation and lactation [79]. Sampling occurred throughout the month of September to intentionally overlap with the peak of pronghorn breeding season (i.e., mid- to late September [61]). Although weaning does not occur until mid-September [80], milk consumption by fawn(s) and output of milk energy are greatly reduced following the first two weeks of lactation [77]. Energy costs for females are reduced following lactation [12], which would explain our findings of lower digestible energy than in late gestation sampling. Female ungulates are typically in the best body condition at the onset of winter when their nutritional demands are lowest [12]. Nevertheless, female pronghorn often spend more time foraging during the breeding season than males [81], likely to meet the energetic demands of weaning, recover from lactation, and prepare for winter migration.

We found weak or negative relationships between maternal nutrition (i.e., FN and DAPA) and maternal stress (i.e., FGMs) during the breeding season, except for a significant positive relationship between FN and FGM in 2019 (Figure 3 and Figure 4). One limitation of FGM estimates is that this parameter represents an integrated measure of secretion over 12–24 h in ungulates [28,30], and thus an explanation for that pattern may represent the overlap of sample collection and the rut. The onset of the pronghorn rut brings harassment of does by territorial and bachelor bucks, leading to the establishment and maintenance of harems of females [61], possibly eliciting distinct stress responses associated with different male breeding strategies.

Glucocorticoids tend to fluctuate with season, sex, reproductive status, and age of the animal, often peaking during the breeding season [26,67,82]. Wild mammalian species do not always exhibit elevated baseline levels during reproduction [65,83,84,85] because high concentrations of FGMs can have negative effects on the reproductive output of females [22]. In addition, glucocorticoids play a fundamental role in the regulation of energy homeostasis during ‘predictable’ life history stages (e.g., reproduction [21]).

## 5. Conclusions

We believe our study is the first to measure and correlate nutrition and glucocorticoid activity metrics at a temporal scale beyond previous studies, across consecutive population-level life-history stages of free-ranging adult female pronghorn. We were able to monitor temporal patterns in female nutrition and glucocorticoid activity across habitat types and correlate some of these changes with measured changes in diet composition. The fecal indices, together with previously published diet-composition data [44], suggest that adult female pronghorn shift among diets differing in protein and digestible energy content in response to the metabolic demands of life history stages. The results are consistent with, rather than provide direct support for, an income-breeding strategy, given the demonstrated diet-switching between functional groups for consistent consumption of high-quality plants.

We acknowledge that our non-invasive sampling design limited our ability to confirm the reproductive status of individual pronghorn; however, we feel these results will assist wildlife managers in further understanding relationships between physiological parameters and how pronghorn meet the metabolic requirements of life history stages. Additionally, we believe this is an important topic that enhances knowledge of the management of wildlands for pronghorn.

## Data Availability

The dataset in this study can be requested from the corresponding author.
